# Effects of TiC, TiH_2_, Al, and Carbon on Production of Ti_3_AlC_2_ by Self-Sustaining Combustion Synthesis

**DOI:** 10.3390/ma18061293

**Published:** 2025-03-14

**Authors:** Chun-Liang Yeh, Yu-Ting Chen

**Affiliations:** Department of Aerospace and Systems Engineering, Feng Chia University, Taichung 40724, Taiwan

**Keywords:** Ti_3_AlC_2_, combustion synthesis, excess Al, TiC, weight fraction, X-ray diffraction

## Abstract

The production of Ti_3_AlC_2_ was investigated by self-propagating high-temperature synthesis (SHS) using the sample compacts composed of elemental powders with or without TiC and TiH_2_ additions. The influence of Al, carbon, TiC, and TiH_2_ was explored on the combustion sustainability, combustion velocity and temperature, and phase composition and microstructure of the product. The experimental results indicated that the elemental sample with an Al-excess composition increased the combustion velocity and improved the formation of Ti_3_AlC_2_, but the sample with a carbon-deficient composition produced the opposite effect. Although both TiC and TiH_2_ additions decreased combustion exothermicity, an appropriate amount of TiC enhanced the yield of Ti_3_AlC_2_. However, the incomplete decomposition made TiH_2_ unsuitable as a source of Ti and resulted in a low yield of Ti_3_AlC_2_. In this study, the final product containing the highest content of Ti_3_AlC_2_ was synthesized from the Al-excess and TiC-added sample of 2.5Ti + 1.2Al + 1.5C + 0.5TiC, and the product was composed of 89.3 wt.% Ti_3_AlC_2_, 5.9 wt.% Ti_2_AlC, and 4.8 wt.% TiC. A reaction mechanism was proposed for the formation of Ti_3_AlC_2_ by SHS, which involved three exothermic reaction steps sequentially producing TiC, Ti_2_AlC, and Ti_3_AlC_2_. The as-synthesized Ti_3_AlC_2_ grains were in the shape of thin platelets with a thickness of about 1.0 μm, and a layered structure formed by closely stacked platelets was clearly observed.

## 1. Introduction

MAX phases are a family of layered hexagonal carbides and nitrides designated by a chemical formula of M*_n_*_+1_AX*_n_* with *n* = 1–4, where M is an early transition metal, A is an A-group element (mostly IIIA and IVA elements), and X is either carbon or nitrogen [1]. Ti_3_AlC_2_ is a member of the MAX family in the Ti-Al-C system and was first discovered by Pietzka and Schuster [2] through the sintering of a cold-compacted powder mixture of Ti, TiAl, Al_4_C_3,_ and carbon in a hydrogen atmosphere at 1300 °C for 20 h. Ti_3_AlC_2_ exhibits a unique combination of excellent properties of both metals and ceramics due to its covalent M-X bond and metallic M-A bond [3]. Like metals, Ti_3_AlC_2_ is thermally and electrically conductive, easily machinable with conventional high-speed tools, and resistant to thermal shock; like ceramics, Ti_3_AlC_2_ possesses low density, high strength and modulus, high melting point, and thermal stability, and good oxidation resistance with self-healing capability [4,5,6,7,8,9]. Recently, Ti_3_AlC_2_ has been a precursor for the preparation of Ti_3_C_2_T_x_ MXene by means of HF and LiF-HCl etching approaches to selectively remove the A layer from the parent MAX phase [10,11,12].

The main characteristic of MAX phases is their unique combination of properties, filling the gap between ceramics and metals. In addition to being used as a replacement for graphite at high temperatures and heating elements, Ti_3_AlC_2_ and other MAX phases, such as Ti_3_SiC_2_, Ti_2_AlC, and Cr_2_AlC, have numerous potential applications [13], including structural materials for high-temperature applications [14,15], protective coatings and bond coats for gas turbines [16,17], accident tolerant fuel cladding in nuclear power plants [18], solar receiver in concentrated solar power systems [19], electrical contacts [20,21], and catalyst [22,23]. The discovery of a new family of two-dimensional materials, MXenes, for which MAX phases are currently the only precursor, has further enhanced the importance of producing this class of materials [24].

Many fabrication methods have been adopted to prepare Ti_3_AlC_2_, including hot isostatic pressing (HIP) [4,7], spark plasma sintering (SPS) [25,26,27,28], molten salt synthesis [29,30], pressureless synthesis [31,32], mechanochemical synthesis [33], and combustion synthesis or self-propagating high-temperature synthesis (SHS) [34,35,36,37,38]. Moreover, a variety of raw materials with different compositional ratios have been employed in the reactant mixtures. Tzenov and Barsoum [4] conducted the synthesis and characterization of polycrystalline Ti_3_AlC_2_ from the Ti-Al_4_C_3_-C powder mixture with Ti:Al:C = 3:1.1:1.8 by HIP at 70 MPa and 1400 °C for 16 h. The HIP approach has the limitations of high energy consumption and long treatment time. As a modification, Wang and Zhou [7] fabricated fully-dense polycrystalline Ti_3_AlC_2_ by the solid–liquid reaction of a mixture consisting of Ti, Al, and graphite powders and simultaneous in situ hot pressing at 1500 °C and 25 MPa for 5 min and subsequently annealing at 1200 °C for 20 min. With the advantages of fast heating and cooling rates and dense products, the SPS technique was employed by Gao et al. [25] to prepare Ti_3_AlC_2_ at 1200–1400 °C for 10–60 min and to investigate the effects of the composition proportion of reactant powders, heating temperature, and holding time. The starting materials included Ti, Al, and graphite powders with three molar ratios of Ti:Al:C = 3:1:2, 3:1.1:1.8, and 3:1.2:2. Results showed that Ti_3_AlC_2_ with a high purity of 97.23 wt.% was synthesized from the Al-excess sample of Ti:Al:C = 3:1.2:2 heated at 1300 °C for 60 min. The decrease in the amount of graphite at Ti:Al:C = 3:1.1:1.8 led to the formation of multiple phases in the end products, including Ti_3_AlC_2_, Ti_2_AlC, Ti_3_AlC, Ti_3_Al, and TiC [25]. The content of Al in the elemental powder mixtures of 3Ti/xAl/2C with x = 1, 1.1, 1.2, and 1.3 was studied by Yang et al. [26] on the formation of Ti_3_AlC_2_ through SPS at 1050–1150 °C for 10 min, indicating that the Al-rich sample with Ti:Al:C = 3:1.2:2 yielded Ti_3_AlC_2_ with the highest purity of 99.4 wt.%. Yunus et al. [27] also reported that Ti_3_AlC_2_ synthesized by SPS from the sample with an Al-excess composition of Ti:Al:C = 3:1.2:2 had higher purity than that from the sample with a carbon-deficient composition of Ti:Al:C = 3:1:1.8. It was believed that 20% excess Al in the starting powder mixture could compensate for the evaporation loss of Al during high-temperature sintering because of the low melting temperature and high vapor pressure of Al [25,26,27].

In addition to elemental powders, TiC and TiH_2_ were adopted as raw materials in many studies. Peng et al. [31] synthesized Ti_3_AlC_2_ by heating 2TiC/Al/C powders at 1300–1400 °C for 15–30 min. Yang et al. [32] fabricated porous Ti_3_AlC_2_ by reactive synthesis of TiH_2_, Al, and graphite powers with different molar ratios of 3:1:2, 3:1.2:2, and 3:1.4:2. It was reported that the deposition of TiH_2_ at 700 °C yielded Ti, which reacted with molten Al at 800–1000 °C to form TiAl_3_, TiAl, and Ti_3_Al. Subsequent reactions among Ti, Ti-Al intermetallic compounds, and graphite produced TiC, Ti_3_AlC, Ti_2_AlC, and Ti_3_AlC_2_ at 1100–1300 °C. Additionally, Yang et al. [32] showed that the sample containing excess Al with a molar ratio of TiH_2_:Al:C = 3:1.2:2 was beneficial to the synthesis of Ti_3_AlC_2_.

Another approach for preparing the MAX phases, such as Ti_2_AlC, Ti_2_SnC, Ti_3_AlC_2_, and Ti_3_SiC_2_, is the SHS technique, where the exothermic reaction is exploited [10,39]. Although the pure MAX phase is challenging to achieve, the SHS method still has a few practical advantages, such as high energy release, low energy consumption, short reaction time, cost-effectiveness, small grain size, and fast cooling rate. Zhou et al. [34] synthesized Ti_2_AlC and Ti_3_AlC_2_ by SHS using elemental powders of Ti, Al, and carbon black. Ge et al. [35] obtained Ti_3_AlC_2_ by combustion synthesis from TiC-added Ti/Al/C powder mixtures and pointed out that TiC as an additive facilitated the synthesis of Ti_3_AlC_2_. The reaction mechanism involved the dissolution of TiC into the Ti-Al melt and subsequent precipitation of the Ti_3_AlC_2_ phase that grew into a layered morphology at a temperature lower than 1450 °C. The effects of TiC and Al_4_C_3_ as the reactants on the formation of Ti_3_AlC_2_ by SHS were investigated from powder mixtures of Ti/Al/C/TiC and Ti/Al/C/Al_4_C_3_, both of which remained in an exactly stoichiometric ratio of Ti:Al:C = 3:1:2 [36,37]. The end products were composed of Ti_3_AlC_2_, Ti_2_AlC, and TiC. Both the TiC and Al_4_C_3_ additions were found to improve the formation of Ti_3_AlC_2_. Compared to the 80 wt.% Ti_3_AlC_2_ produced by the Al_4_C_3_-added sample, the product containing 84.6 wt.% Ti_3_AlC_2_ was synthesized from the TiC-added sample [36,37]. Akhlaghi et al. [38] prepared Ti_3_AlC_2_ from mechanically-activated Ti, Al, and graphite powders at Ti:Al:C = 3:1:2 by both SHS and thermal explosion techniques. Due to more Al loss during the SHS process, the yield of Ti_3_AlC_2_ (60 wt.%) produced by SHS was lower than that (85 wt.%) obtained by combustion synthesis in the thermal explosion mode. So far, the purity of Ti_3_AlC_2_ produced by the SHS method was not as high as that obtained by other methods, like HIP and SPS. Moreover, the green samples with off-stoichiometric ratios, such as Al-rich and carbon-lean compositions, have rarely been adopted by the SHS studies.

The goal of producing Ti_3_AlC_2_ using the SHS methods was to develop an efficient and economical pathway for the industrial preparation of this MAX phase. The SHS method takes advantage of a self-sustaining exothermic reaction and has the merits of energy efficiency, productivity and self-purification behavior, affordable precursors, and simplicity of the equipment used. SHS also provides a vast variety of product shapes and dimensions, from films to porous structures, powders, or high-density bulks. Moreover, easy scale-up is characteristic of combustion synthesis [39]. The specific objective of the present study was to investigate the effects of different reactant combinations and proportions on the formation of Ti_3_AlC_2_ by combustion synthesis in the SHS mode. Test samples formulated by the elemental powders were conducted with or without TiC and TiH_2_ additions. The influence of Al-excess and carbon-deficient compositions was explored. In this study, the propagation velocity and temperature of the self-sustaining combustion wave were measured. The microstructure and phase composition of the synthesized products were examined. In addition, the reaction mechanism of forming Ti_3_AlC_2_ through combustion synthesis was proposed.

## 2. Materials and Methods

The starting materials of this study included Ti (Alfa Aesar, Ward Hill, MA, USA, <45 μm, and 99.8%), Al (Alfa Aesar, Ward Hill, MA, USA, <45 μm, and 99.7%), carbon black (Showa Chemical Co., Tokyo, Japan), TiC (Aldrich Chemical, Burlington, MA, USA, <15 μm, and 99%), and TiH_2_ (Strem Chemicals, Newburyport, MA, USA, <75 μm, and 98%). Three mixture combinations of powders were formulated as expressed in Equations (1)–(3).

(1) $$3Ti+mAl+nC\to {Ti}_{3}AlC_{2}$$

(2) $$\left(3-x\right)Ti+1.2Al+\left(2-x\right)C+xTiC\to {Ti}_{3}AlC_{2}$$

(3) $$\left(3-y\right)Ti+1.2Al+2C+yTiH_{2}\to {Ti}_{3}AlC_{2}+yH_{2}$$

The stoichiometric parameters, *m* and *n*, of Equation (1) represent the number of moles of Al and carbon, respectively, in the elemental powder mixture. The value of *m* varying from 1.0 to 1.3 was considered to examine the effect of excess Al, i.e., the Al-rich composition. The effect of deficient carbon (i.e., the carbon-lean composition) was studied by changing the value of *n* between 1.8 and 2.0. For the TiC- and TiH_2_-added reaction systems of Equations (2) and (3), the powder mixtures were formulated under excess Al by 20%. The parameters *x* and *y* signify the number of moles of TiC- and TiH_2_ in Equations (2) and (3), respectively. In this study, the amount of TiC addition varied from *x* = 0.1 to 0.5 and that of TiH_2_ from *y* = 0.1 to 0.4. Table 1 summarizes 19 different samples conducted by this study with their compositions specified by stoichiometric parameters of Equations (1)–(3).

Reactant powders were dry mixed by a horizontal jar mill in the air atmosphere for 4 h and alumina grinding balls with a diameter of 1 mm were used. The ball-to-powder ratio was 7:1. PTFE (Teflon) milling jars were used. Teflon milling jars are non-sticky and have been widely used because of their minimal contamination and easy-to-clean benefits. Then, the powder mixture was uniaxially compressed into a cylindrical shape with a diameter of 7 mm, a height of 12 mm, and a relative density of 50%. The SHS experiment was performed in a windowed stainless-steel combustion chamber filled with Ar at 0.25 MPa. The viewing windows were made of quartz. The power compact was ignited on the top surface by a heated tungsten coil. The propagation velocity of the self-sustaining combustion wave (V_f_) was determined from the time sequence of recorded images. The combustion temperature was measured by a bare-wire thermocouple (Pt/Pt-13%Rh) with a bead size of 125 μm. Details of the experimental setup and methods of approach have been reported elsewhere [40,41]. The phase composition and microstructure of the synthesized products were analyzed using an X-ray diffractometer (XRD, Bruker D2 Phaser, Karlsruhe, Germany) with CuK_α_ radiation and a scanning electron microscope (SEM, Hitachi, S3000H, Tokyo, Japan), respectively. Based on the XRD pattern, three non-overlapping diffraction peaks were selected to quantitatively determine the weight fractions of Ti_3_AlC_2_, Ti_2_AlC, and TiC in the product according to the following equations [42]:

(4) $$W_{a}=\frac{I_{a}}{I_{a}+0.220I_{b}+0.084I_{c}}$$

(5) $$W_{b}=\frac{I_{b}}{4.545I_{a}+I_{b}+0.382I_{c}}$$

(6) $$W_{c}=\frac{I_{c}}{11.905I_{a}+2.619I_{b}+I_{c}}$$

where *W_a_*, *W_b_*, and *W_c_* are the weight percentages of Ti_3_AlC_2_, Ti_2_AlC, and TiC, respectively. *I_a_*, *I_b_*, and *I_c_* are the integrated intensities of diffraction peaks associated with Ti_3_AlC_2_ (002) at 2θ = 9.5°, Ti_2_AlC (002) at 2θ = 13.0°, and TiC (111) at 2θ = 35.9°, respectively; these three peaks are not overlapped with signals from other phases in the Ti-Al-C ternary system and are appropriate for quantitative phase analysis [42]. The equations are based on the reference intensity ratio method, and the numeric values correspond to the compound corundum factors.

## 3. Results

### 3.1. Combustion Wave Velocity and Combustion Temperature

**Figure 1:**
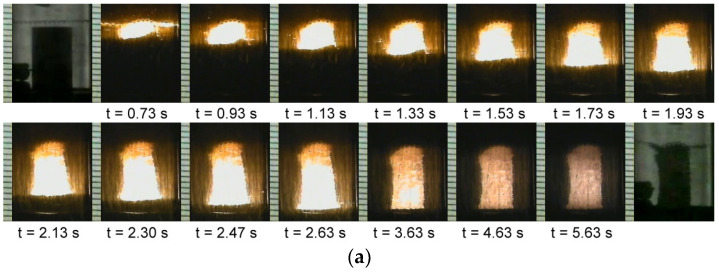

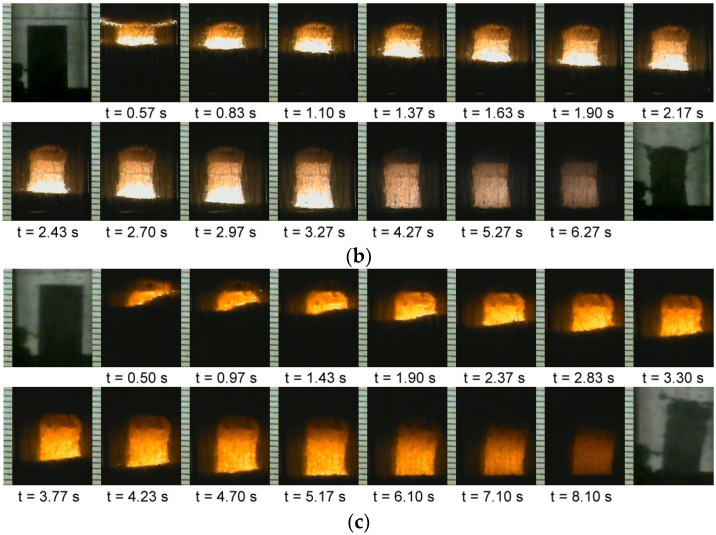
Time sequences of recorded SHS images illustrating the propagation of self-sustaining combustion wave of (**a**) an elemental sample of Ti:Al:C = 3:1:2, (**b**) a TiC-added sample of *x* = 0.3, and (**c**) a TiH_2_-added sample of *y* = 0.3.

The experimental observations of this study showed that the combustion wave velocity and temperature varied with the contents of Al and carbon, as well as with the addition of TiC and TiH_2_. Moreover, the amounts of TiC and TiH_2_ affected the sustainability of the combustion wave. Figure 1a–c illustrates time sequences of the recorded film images showing the propagation of combustion wave along an elemental sample of Ti:Al:C = 3:1:2, a TiC-added sample of *x* = 0.3, and a TiH_2_-added sample of *y* = 0.3. Upon ignition on the top surface of the sample compact, a distinct combustion front formed and traversed lengthwise to the bottom of the sample in a self-sustaining manner. This implies that the synthesis reaction is sufficiently exothermic to maintain self-sustainability. When compared with the elemental powder compact, as revealed in Figure 1a–c, the TiC- and TiH_2_-added samples exhibit a longer flame propagation time and weaker combustion luminosity. That is, the addition of TiC and TiH_2_ could reduce the combustion wave velocity and the degree of combustion exothermicity.

**Figure 2:**
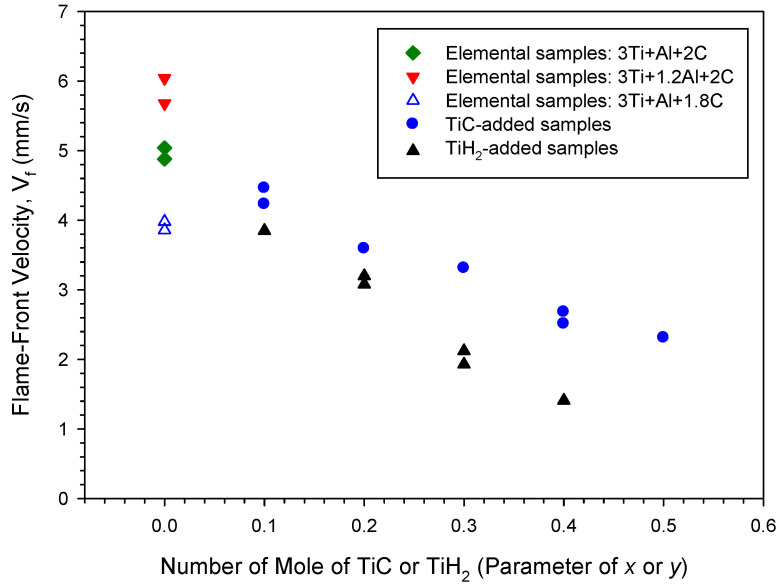
Effects of Al, carbon, TiC, and TiH_2_ on combustion-front velocity of sample compacts.

The variation in the self-sustaining combustion wave velocity with the contents of Al, carbon, TiC, and TiH_2_ is presented in Figure 2. For the elemental samples of Equation (1), the increase in Al accelerated the combustion front, but the decrease in carbon slowed it down. As revealed in Figure 2, the flame-front velocity is around 5 mm/s for the samples of exact stoichiometry at Ti:Al:C = 3:1:2; it increases to 5.7–6.0 mm/s for the samples with excess Al of Ti:Al:C = 3:1.2:2. This could be due to the formation of more molten Al, which facilitated the diffusion of solid reactants and dissolution of TiC into the Ti-Al melt [35]. On the other hand, the combustion velocity decreased to about 3.9 mm/s as the content of carbon was reduced in the samples of Ti:Al:C = 3:1:1.8. It is believed that, as to be discussed later, the combustion wave velocity was governed by the reaction of Ti with carbon. The formation of TiC from the elemental reaction between Ti and carbon is not only the initiation step but the most exothermic reaction of the synthesis process. The carbon-lean sample certainly produced a smaller amount of TiC and released less reaction heat, and, therefore, exhibited a slower combustion wave.

A significant decline in the combustion wave velocity was observed in the TiC- and TiH_2_-added samples. Figure 2 shows the decrease in flame-front velocity from 4.5 to 2.3 mm/s as the TiC content increases from *x* = 0.1 to 0.5, beyond which combustion ceases to propagate and is quenched. This was caused by the dilution effect of TiC on combustion. When the reaction exothermicity was no longer sufficient to maintain the combustion process in a self-propagating mode, the burning was extinguished. As indicated in Figure 2, the decline in combustion velocity was more pronounced for the TiH_2_-added samples, and the flame-front velocity was lowered from 3.9 mm/s at *y* = 0.1 to 1.4 mm/s at *y* = 0.4, and, further than that, combustion failed to be initiated. Because the decomposition of TiH_2_ to produce Ti is a highly endothermic process with Δ*H* = 144.4 kJ/mol [43] and the conversion took place at temperatures above 700 °C [32], this made the TiH_2_-added samples less energetic. Moreover, H_2_ gas formed from the decomposition of TiH_2_ and the release of gaseous hydrogen from the porous powder compact could cause additional heat loss. This explains the substantial decrease in combustion velocity and limited range of combustibility observed in this study for the TiH_2_-added samples. It should be noted that one or two experiments were conducted for each sample in this study. When two specimens were synthesized, two experimental points were reported for one type of sample. Repeated experiments were performed to verify the reproducibility of the measured data.

**Figure 3:**
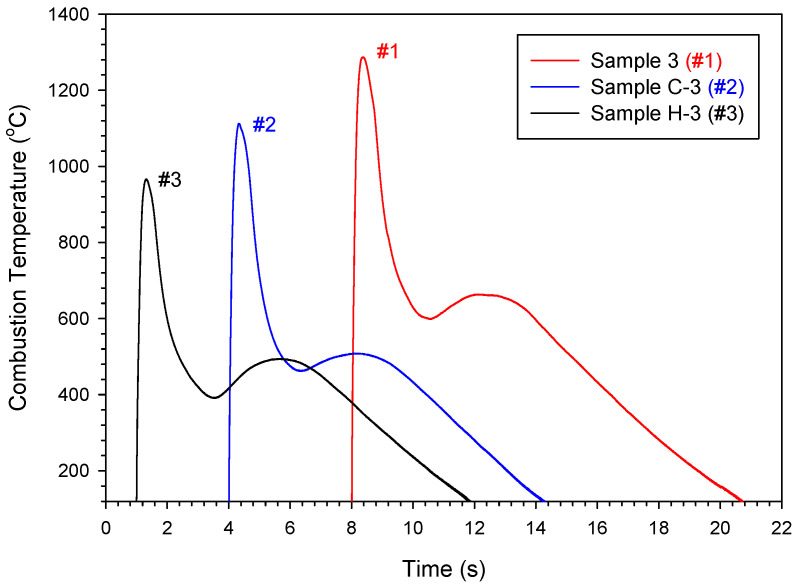
Combustion temperature profiles of an elemental powder compact of Ti:Al:C = 3:1.2:2, a TiC-added sample of *x* = 0.3, and a TiH_2_-added sample of *y* = 0.3.

The propagation behavior of the combustion wave in the SHS process is subject to the layer-by-layer heat transfer from the reaction zone to the unburned region, and the combustion temperature plays an important role in the speed of the combustion front [44,45]. Figure 3 depicts three typical combustion temperature profiles measured from different types of sample compacts. All profiles exhibited a sharp temperature upsurge followed by an instant descent, which is typical of the SHS reaction that features a fast combustion wave and a thin reaction zone. The peak value was considered as the combustion-front temperature (*T*_c_). After the passage of the combustion wave, a bump-shaped rise succeeded by a slow decline in temperature was shown. The presence of the bump-shaped contour might signify the occurrence of a volumetric reaction after the passage of the combustion front. Because it was difficult for the synthesis reaction to complete within a thin and rapid combustion front, the reaction continued in a bulk fashion. Among the three samples in Figure 3, the Al-rich elemental sample of Ti:Al:C = 3:1.2:2 shows the highest *T*_c_ of 1287 °C. The lower values of *T*_c_ of 1112 °C and 966 °C were detected from the TiC-containing sample of *x* = 0.3 and TiH_2_-added sample of *y* = 0.3, respectively. The plateau temperature associated with the bump-shaped region was also affected by the addition of TiC and TiH_2_ and varied between 500 °C and 660 °C.

**Figure 4:**
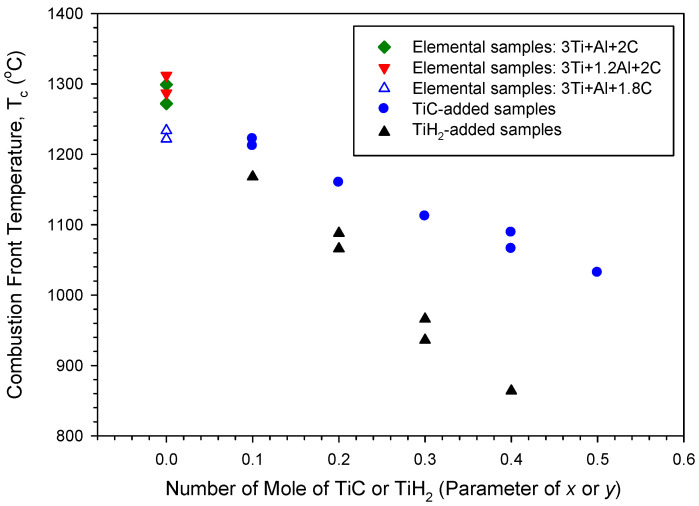
Effects of Al, carbon, TiC, and TiH_2_ on combustion-front temperature of sample compacts.

Figure 4 presents the variation in combustion-front temperature with the sample composition. For the elemental powder compacts, it was reported that excess Al had little influence on *T*_c_, but a lack of carbon lowered *T*_c_ to some extent. That is, *T*_c_ varied between 1280 °C and 1310 °C for the samples of Ti:Al:C = 3:1:2 and 3:1.2:2 and was about 1230 °C for the sample of Ti:Al:C = 3:1:1.8. Due to the dilution effect of TiC and endothermic decomposition of TiH_2_, as mentioned above, Figure 4 reveals that the increase in TiC and TiH_2_ additions decreases the combustion-front temperature. When compared to *T*_c_ varied between 1222 °C and 1032 °C for the TiC-added samples with *x* = 0.1–0.5, lower combustion-front temperatures in the range from 1168 °C to 870 °C were detected for the TiH_2_-added samples with *y* = 0.1–0.4. This confirms that the combustion of the TiC-added sample is more exothermic than that of the TiH_2_-containing sample. In this study, the possible random error in the combustion velocity and temperature might be caused by the non-planar combustion front and the deformation of the sample compact.

### 3.2. Phase Composition and Microstructure Analyses of Synthesized Products

**Figure 5:**
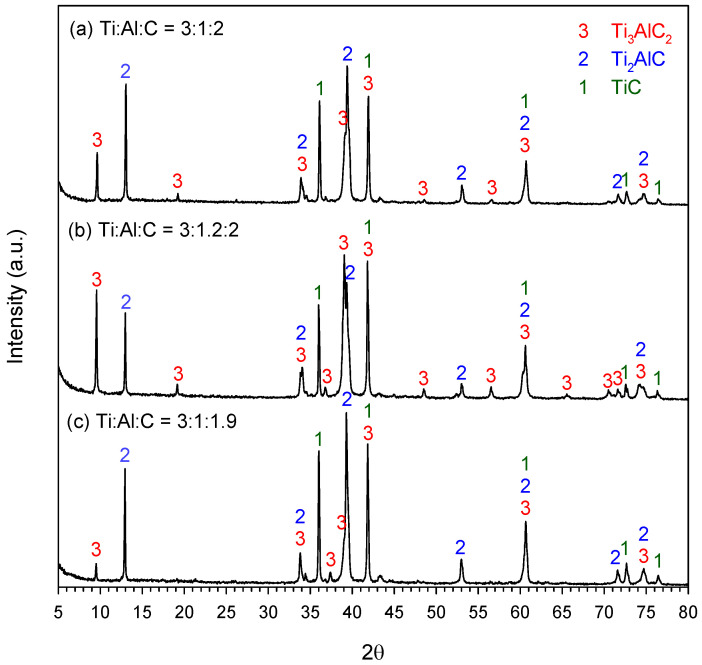
XRD patterns of the synthesized products from elemental powder compacts with starting compositions of (**a**) Ti:Al:C = 3:1:2, (**b**) Ti:Al:C = 3:1.2:2, and (**c**) Ti:Al:C = 3:1:1.9.

Figure 5a–c presents the XRD patterns of the products synthesized from three elemental powder compacts of Equation (1) with different compositions. Final products composed of three constituent phases, Ti_3_AlC_2_, Ti_2_AlC, and TiC, were found. Standard JCPDS cards 52-0875, 29-0095, and 65-0971 were used to identify Ti_3_AlC_2_, Ti_2_AlC, and TiC, respectively. Due to the inherently complex nature of the Ti-Al-C ternary system, many transient intermetallics and carbides, such as Ti_3_Al, TiAl, TiC, Ti_3_AlC, and Ti_2_AlC, were previously detected while synthesizing Ti_3_AlC_2_ [26,27,32,35,46]. Based on the relative magnitude of signature diffraction peaks of Ti_3_AlC_2_ at 2θ = 9.5°, Ti_2_AlC at 2θ = 13.0°, and TiC at 2θ = 35.9° adopted in Equations (4)–(6), it can be seen that the sample with excess Al of Ti:Al:C = 3:1.2:2 shown in Figure 5b produced more Ti_3_AlC_2_ than the exact-stoichiometry sample of Ti:Al:C = 3:1:2 in Figure 5a. Moreover, it is apparent from Figure 5c that the yield of Ti_3_AlC_2_ was considerably reduced in the carbon-lean sample of Ti:Al:C = 3:1:1.9. The XRD patterns of other products of Equation (1) can be found in Supplementary Materials of this paper.

The weight percentages of Ti_3_AlC_2_, Ti_2_AlC, and TiC of the products obtained from the elemental powder compacts of Equation (1) were calculated using Equations (4)–(6) and summarized in Table 2. For the exactly-stoichiometric sample of Ti:Al:C = 3:1:2, the resulting product was composed of 56.3 wt.% Ti_3_AlC_2_, 31.5 wt.% Ti_2_AlC, and 12.2 wt.% TiC. The addition of excess Al enhanced the production of Ti_3_AlC_2_ to a great extent, and the highest yield reaching 80.3 wt.% was achieved by the sample of Ti:Al:C = 3:1.2:2, within which a substantial decrease in Ti_2_AlC and TiC, respectively, to about 12.9 wt.% and 6.8 wt.%, was found. Table 1 points out that the optimum amount of excess Al is 20%, which agrees with the findings of many previous studies [25,26,27,32]. It has been proposed that excess Al in the starting powder mixture could compensate for the evaporation loss of Al during the high-temperature synthesis process because of the low melting temperature and high vapor pressure of Al [25,26,27]. The other benefit of excess Al could be associated with the formation of the TiAl phase, which has been considered one of the intermediates in the synthesis of Ti_3_AlC_2_ [26,27,32,46]. According to the Ti-Al phase diagram [47,48], the TiAl phase has a wide homogeneity range from 49 at.% Al to about 66 at.% Al, depending on the temperature. Specifically, the homogeneity ratio is 49 at.% Al at 500 °C and is gradually broadened to 66 at.% Al at 1400 °C. This suggests that an Al-rich sample is beneficial to produce TiAl.

On the other hand, Table 2 indicates relatively low yields of Ti_3_AlC_2_ from the samples deficient in carbon. The weight fraction of Ti_3_AlC_2_ of about 30 wt.% was produced by the carbon-lean samples of Ti:Al:C = 3:1:1.9 (i.e., *m* = 1 and *n* = 1.9). Although the content of Ti_3_AlC_2_ was improved to 49 wt.% by extra Al addition (*m* = 1.2), this value was still lower than that of the carbon-sufficient samples (*n* = 2). The production of Ti_3_AlC_2_ was further worsened for the samples containing a lesser amount of carbon of *n* = 1.8, for which the resulting products consisted only of 10–13 wt.% Ti_3_AlC_2_ and were dominated by Ti_2_AlC. The low level of production of Ti_3_AlC_2_ was largely due to a lack of TiC to convert Ti_2_AlC to Ti_3_AlC_2_. Based on the experimental results of elemental powder compacts presented in Table 1, an optimum atomic ratio of Ti:Al:C = 3:1.2:2 was attained, and this proportion was adopted in the TiC- and TiH_2_-added samples of Equations (2) and (3).

The reaction mechanism of producing Ti_3_AlC_2_ through combustion synthesis in the SHS mode was proposed based on the nature of self-sufficiency in energy of the SHS process. Three key exothermic reactions, as expressed in Equations (7)–(9), were involved. The heat of reaction (Δ*H*_r_) and adiabatic temperature (*T*_ad_) of Equations (7)–(9) were calculated from the thermochemical data [33,34,49] and energy balance equation [50]. The SHS process relies on a highly exothermic reaction to maintain self-sustainability. It is proposed that Equation (7), which relates to forming TiC from the reaction of Ti with carbon, acts as the initiation step, which is adequately energetic and has an adiabatic temperature of up to 3120 K. Such a high temperature justifies the self-sustainability of the synthesis process. Unlike other fabrication routes, which detected intermetallic phases of Ti_3_Al and TiAl in the end products, the subsequent reaction could proceed with another exothermic reaction of Equation (8), which is the dissolution of TiC and Ti into the Al melt. Finally, Ti_3_AlC_2_ was produced through Equation (9), where the interaction between TiC and Ti_2_AlC occurred. Although Equations (8) and (9) are not as energetic as Equation (7), both reactions are exothermic. It is believed that Equation (7) is the most important heat-releasing step in the reaction mechanism and governs the propagation of the high-temperature combustion front. Equations (8) and (9) are responsible for the volumetric reaction.

(7) $$Ti+C\to TiC{(∆H}_{r}=-184.1\;kJ/mol,\;T_{ad}=3120\;K)$$

(8) $$TiC+Ti+Al\to {Ti}_{2}AlC{(∆H}_{r}=-74.3\;kJ/mol,\;T_{ad}=1204\;K)$$

(9) $$TiC+{Ti}_{2}AlC\to {Ti}_{3}AlC_{2}{(∆H}_{r}=-106.65\;kJ/mol,\;T_{ad}=1020\;K)$$

**Figure 6:**
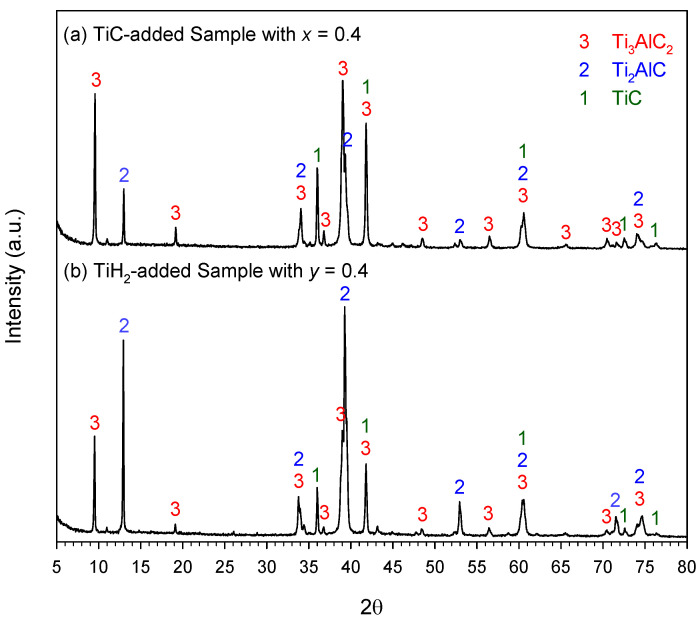
XRD patterns of the synthesized products from (**a**) TiC-added sample with *x* = 0.4 and (**b**) TiH_2_-added sample with *y* = 0.4.

**Figure 7:**
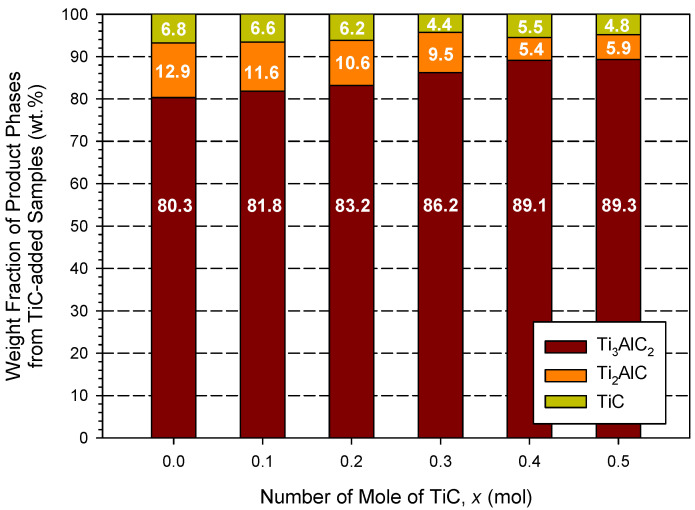
Weight fractions of Ti_3_AlC_2_, Ti_2_AlC, and TiC in synthesized products from TiC-added samples of Equation (2).

The XRD spectra presented in Figure 6a,b are associated with the final products obtained from TiC- and TiH_2_-added samples of *x* = 0.4 and *y* = 0.4, respectively. Like the elemental samples, the XRD analysis identified three constituent phases, Ti_3_AlC_2_, Ti_2_AlC, and TiC. Based on the signature peaks of these three phases, Figure 6a indicates Ti_3_AlC_2_ as being the major phase in the product of the TiC-added sample, while Figure 6b signifies Ti_2_AlC as dominating over others in the TiH_2_-added sample. The XRD patterns of other products of Equations (1) and (2) can be found in Supplementary Materials of this paper. The weight fractions of Ti_3_AlC_2_, Ti_2_AlC, and TiC in the products of the TiC-added samples are reported in Figure 7. An improvement in Ti_3_AlC_2_ production by increasing the amount of TiC addition was shown, and the highest yield of Ti_3_AlC_2_, reaching 89.3 wt.%, was achieved by the sample containing a TiC of *x* = 0.5, where the contents of Ti_2_AlC and TiC were as low as 5.9 and 4.8 wt.%, respectively. A comparable composition consisting of 89.1 wt.% Ti_3_AlC_2_, 5.4 wt.% Ti_2_AlC, and 5.5 wt.% TiC was obtained from the TiC-added sample of *x* = 0.4. This confirms the positive effect of TiC addition, which benefits the degree of completeness of Equation (9).

**Figure 8:**
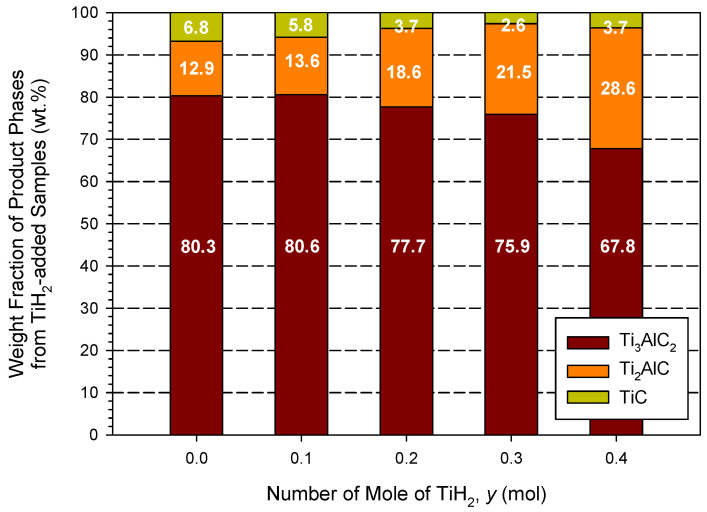
Weight fractions of Ti_3_AlC_2_, Ti_2_AlC, and TiC in synthesized products from TiH_2_-added samples of Equation (3).

Figure 8 reveals the weight fractions of constituent phases of the TiH_2_-added samples. The product composition of the sample containing a TiH_2_ of *y* = 0.1 was almost not affected by such a small amount of TiH_2_ and contained 80.6 wt.% Ti_3_AlC_2_, 13.6 wt.% Ti_2_AlC, and 5.8 wt.% TiC. With the increase in TiH_2_, a decrease in Ti_3_AlC_2_ along with an increase in Ti_2_AlC were found. As shown in Figure 8, the sample of *y* = 0.4 produced 67.8 wt.% Ti_3_AlC_2_, 28.6 wt.% Ti_2_AlC, and 3.7 wt.% TiC. This could be caused by the incomplete decomposition of TiH_2_ due to a decrease in combustion temperature with increasing TiH_2_. The thermal decomposition of TiH_2_ was to provide Ti for the synthesis reaction. A lack of Ti in the TiH_2_-added samples could lower the amount of TiC and hinder the phase conversion from Ti_2_AlC to Ti_3_AlC_2_.

**Figure 9:**
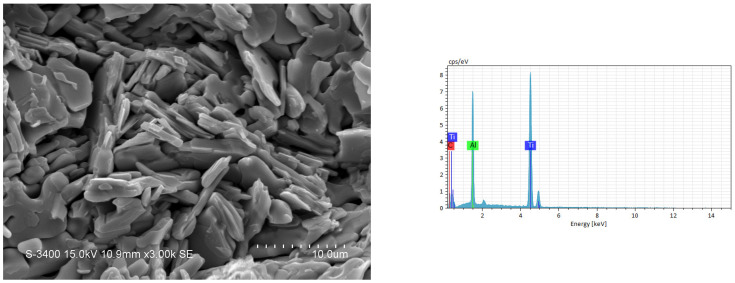
SEM image and EDS spectrum of a synthesized product from the elemental powder compact of Equation (1) with Ti:Al:C = 3:1.2:2 (*m* = 1.2 and *n* = 2).

**Figure 10:**
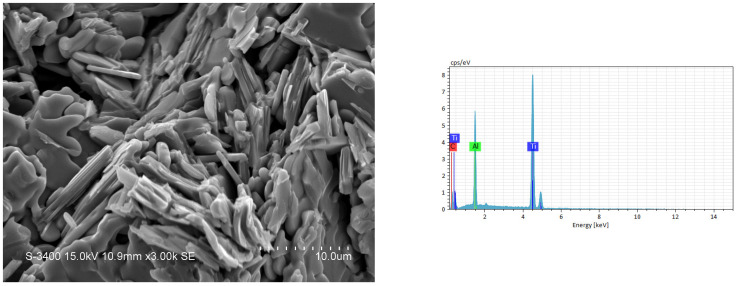
SEM image and EDS spectrum of a synthesized product from TiC-added sample of Equation (2) with *x* = 0.5.

**Figure 11:**
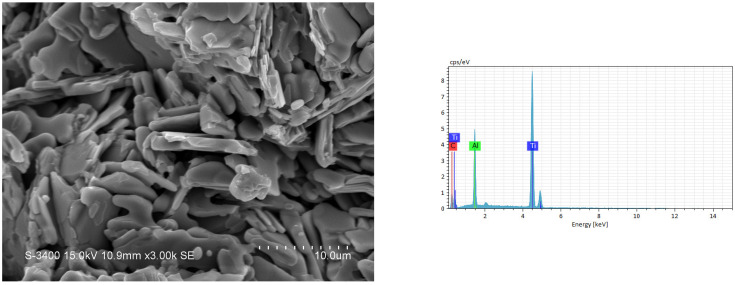
SEM image and EDS spectrum of a synthesized product from TiH_2_-added sample of Equation (3) with *y* = 0.3.

The SEM image and EDS spectrum illustrated in Figure 9 reveal the microstructure of the fracture surface and the atomic ratio of the synthesized product from an elemental powder compact with excess Al of Ti:Al:C = 3:1.2:2. Ti_3_AlC_2_ grains in a platelet shape were produced, and a layered microstructure typical of the MAX ternary carbide was clearly seen. The Ti_3_AlC_2_ platelets were 5–8 μm in size, 1 μm in thickness, and randomly staggered. The atomic ratio of Ti:Al:C = 52.4:17.8:29.8 obtained from the EDS spectrum of Figure 9 matched well with Ti_3_AlC_2_. Figure 10 shows the microstructure and EDS element spectrum of the product synthesized from the TiC-added sample with *x* = 0.5. Plate-like Ti_3_AlC_2_ grains were closely interlocked, and some of them were packed into a laminated configuration. An atomic ratio of Ti:Al:C = 50.1:15.1:34.8 was deduced from the EDS analysis of Figure 10, confirming the formation of Ti_3_AlC_2_. The SEM and EDS results associated with the product synthesized from the TiH_2_-added sample of *y* = 0.3 are shown in Figure 11. Similar to those in Figure 9 and Figure 10, plate-like Ti_3_AlC_2_ grains were observed. The atomic ratio of Ti:Al:C also matched well with Ti_3_AlC_2_. Table 3 lists the atomic ratios of constituent elements deduced from the EDS shown in Figure 9, Figure 10 and Figure 11. Based on the SEM examination, it is believed that the morphology of the synthesized products is essentially not affected by the types of samples used in this study.

## 4. Conclusions

The formation of Ti_3_AlC_2_ was investigated by the SHS method using the sample compacts composed of elemental powders with or without TiC and TiH_2_ additions. The experimental results showed that, for the elemental powder compacts, an Al-excess composition increased the combustion rate and improved the formation of Ti_3_AlC_2_, but a carbon-deficient composition decreased the combustion temperature and deteriorated the yield of Ti_3_AlC_2_. This was because excess Al could compensate for the evaporation loss of Al during the high-temperature synthesis process. A lack of carbon reduced the formation of TiC, which is a highly exothermic phase and plays an important role in the evolution of Ti_3_AlC_2_. An optimum atomic composition of Ti:Al:C = 3:1.2:2 was attained by this study and the elemental powder compact with such a proportion yielded 80.3 wt.% Ti_3_AlC_2_, 12.9 wt.% Ti_2_AlC, and 6.8 wt.% TiC.

With the addition of TiC and TiH_2_, the combustion velocity and temperature were decreased. The product analysis indicated that the addition of TiC enhanced the formation of Ti_3_AlC_2_. The final product of the highest yield was synthesized from the sample of 2.5Ti + 1.2Al + 1.5C + 0.5TiC and it consisted of 89.3 wt.% Ti_3_AlC_2_, 5.9 wt.% Ti_2_AlC, and 4.8 wt.% TiC. On the other hand, TiH_2_, as the reactant, produced a negative effect on the synthesis of Ti_3_AlC_2_, due most likely to the incomplete decomposition of TiH_2_. As a result, the TiH_2_-added sample of 2.6Ti + 1.2Al + 2C + 0.4TiH_2_ yielded only 67.8 wt.% Ti_3_AlC_2_. The as-synthesized Ti_3_AlC_2_ grains exhibited a thin plate-like shape, and the platelets were closely stacked into a laminated configuration, which is typical of the MAX-phase microstructure. Moreover, the atomic proportion of platelet grains deduced from the EDS matched well with Ti_3_AlC_2_.

## Figures and Tables

**Table 1:** Sample compositions of the powder compacts conducted in this study.

Combustion Systems	Sample No.	Parameters
Equation (1): Elemental samples	1	*m* = 1.0, *n* = 2.0
	2	*m* = 1.1, *n* = 2.0
	3	*m* = 1.2, *n* = 2.0
	4	*m* = 1.3, *n* = 2.0
	5	*m* = 1.0, *n* = 1.9
	6	*m* = 1.1, *n* = 1.9
	7	*m* = 1.2, *n* = 1.9
	8	*m* = 1.0, *n* = 1.8
	9	*m* = 1.1, *n* = 1.8
	10	*m* = 1.2, *n* = 1.8
Equation (2): TiC-added samples	C-1	*x* = 0.1
	C-2	*x* = 0.2
	C-3	*x* = 0.3
	C-4	*x* = 0.4
	C-5	*x* = 0.5
Equation (3): TiH_2_-added samples	H-1	*y* = 0.1
	H-2	*y* = 0.2
	H-3	*y* = 0.3
	H-4	*y* = 0.4

**Table 2:** Weight percentages of Ti_3_AlC_2_, Ti_2_AlC, and TiC of the final products synthesized from elemental powder compacts of Equation (1) with different compositions.

Sample No. of Equation (1)	Ti:Al:C	Weight Percentage (wt.%)		
		Ti_3_AlC_2_	Ti_2_AlC	TiC
1	3:1:2	56.3	31.5	12.2
2	3:1.1:2	78.0	15.8	6.2
3	3:1.2:2	80.3	12.9	6.8
4	3:1.3:2	71.7	11.3	17.9
5	3:1:1.9	30.2	43.0	26.8
6	3:1.1:1.9	33.5	52.4	14.1
7	3:1.2:1.9	48.9	31.3	19.8
8	3:1:1.8	12.8	38.9	48.3
9	3:1.1:1.8	10.9	75.7	13.4
10	3:1.2:1.8	9.4	80.9	9.7

**Table 3:** Atomic ratio of constituent elements deduced from EDS in Figure 9, Figure 10 and Figure 11.

Element	Atomic Percentage (at.%)		
	Figure 9	Figure 10	Figure 11
C	29.78	34.81	31.26
Al	17.84	15.05	15.14
Ti	52.38	50.14	53.60

## Data Availability

The original contributions presented in this study are included in the article. Further inquiries can be directed to the corresponding author.

## Supplementary Materials

The following supporting information can be downloaded at: https://www.mdpi.com/article/10.3390/ma18061293/s1, Figure S1: XRD patterns of synthesized products from (a) Sample 2, (b) Sample 4, (c) Sample 6, (d) Sample 7, (e) Sample 8, (f) Sample 9, and (g) Sample 10; Figure S2: XRD patterns of synthesized products from (a) Sample C-1, (b) Sample C-2, (c) Sample C-3, and (d) Sample C-5; Figure S3: XRD patterns of synthesized products from (a) Sample H-1, (b) Sample H-2, and (c) Sample C-3.
