# Supplementary material for: Effects of TiC, TiH2, Al, and Carbon on Production of Ti3AlC2 by Self-Sustaining Combustion Synthesis
Source: Materials (Basel). 2025 Mar 14;18(6):1293. doi: 10.3390/ma18061293 (PMC11944232; doi:10.3390/ma18061293)
Supplement: Supplementary file 1 [file materials-18-01293-s001.zip › materials-3514828-supplementary.pdf]

Supplementary File

This file contains the product XRD patterns that are not included in the main content.

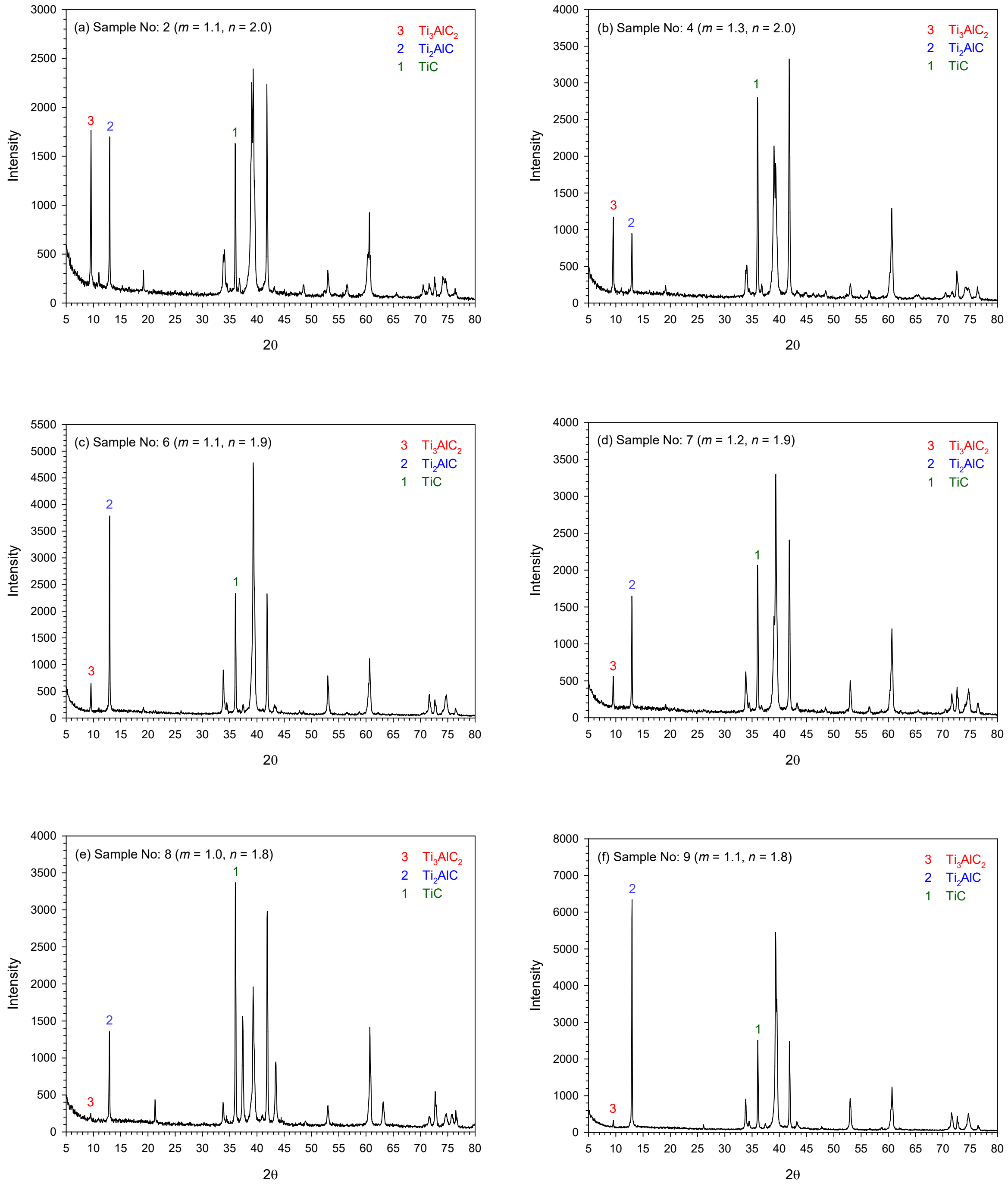

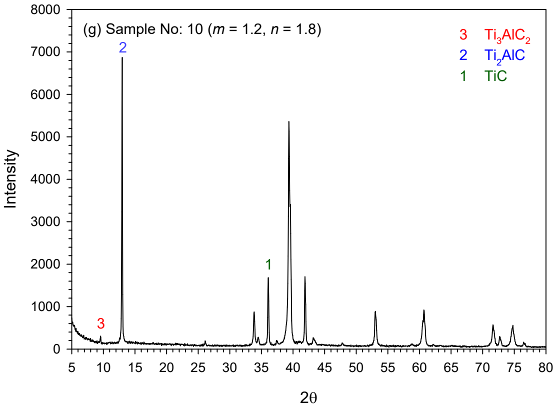

Figure S1. XRD patterns of synthesized products from (**a**) Sample 2, (**b**) Sample 4, (**c**) Sample 6, (**d**) Sample 7, (**e**) Sample 8, (**f**) Sample 9, and (**g**) Sample 10.

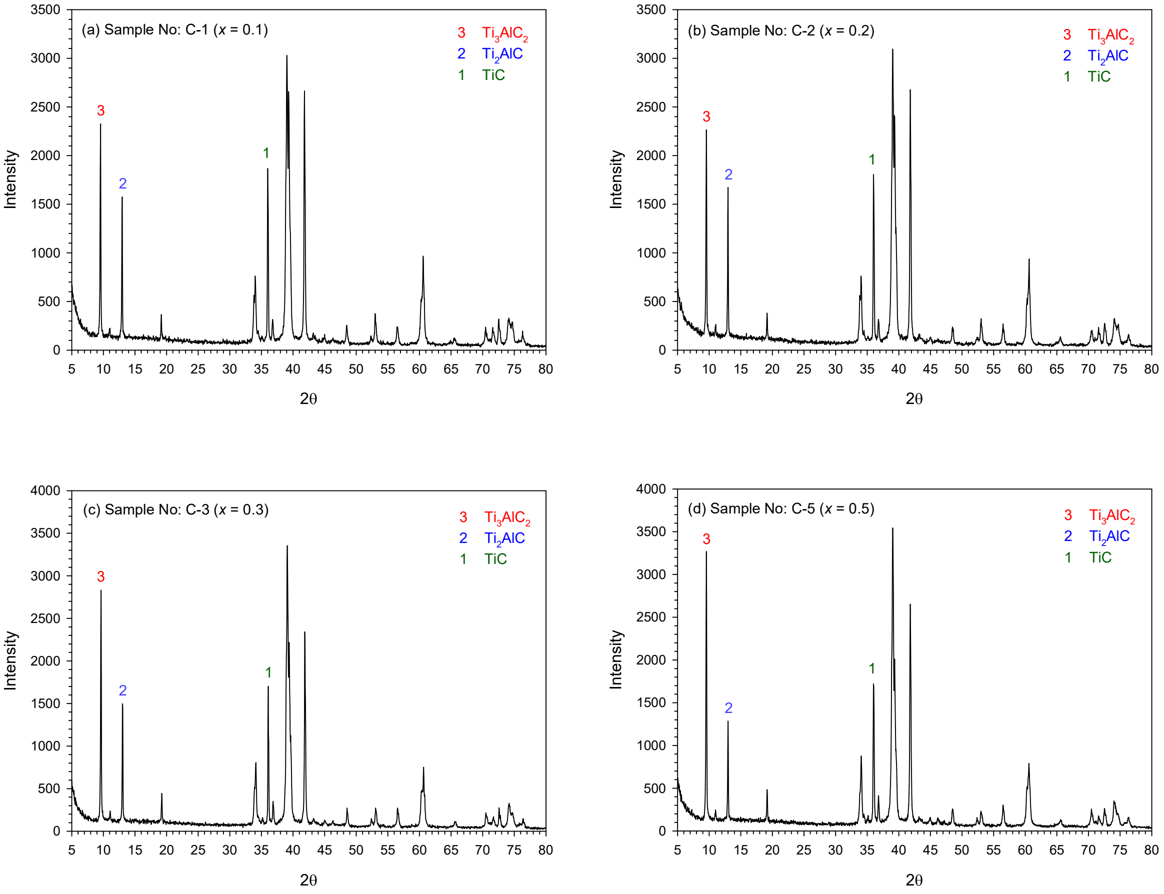

Figure S2. XRD patterns of synthesized products from (**a**) Sample C-1, (**b**) Sample C-2, (**c**) Sample C-3, and (**d**) Sample C-5.

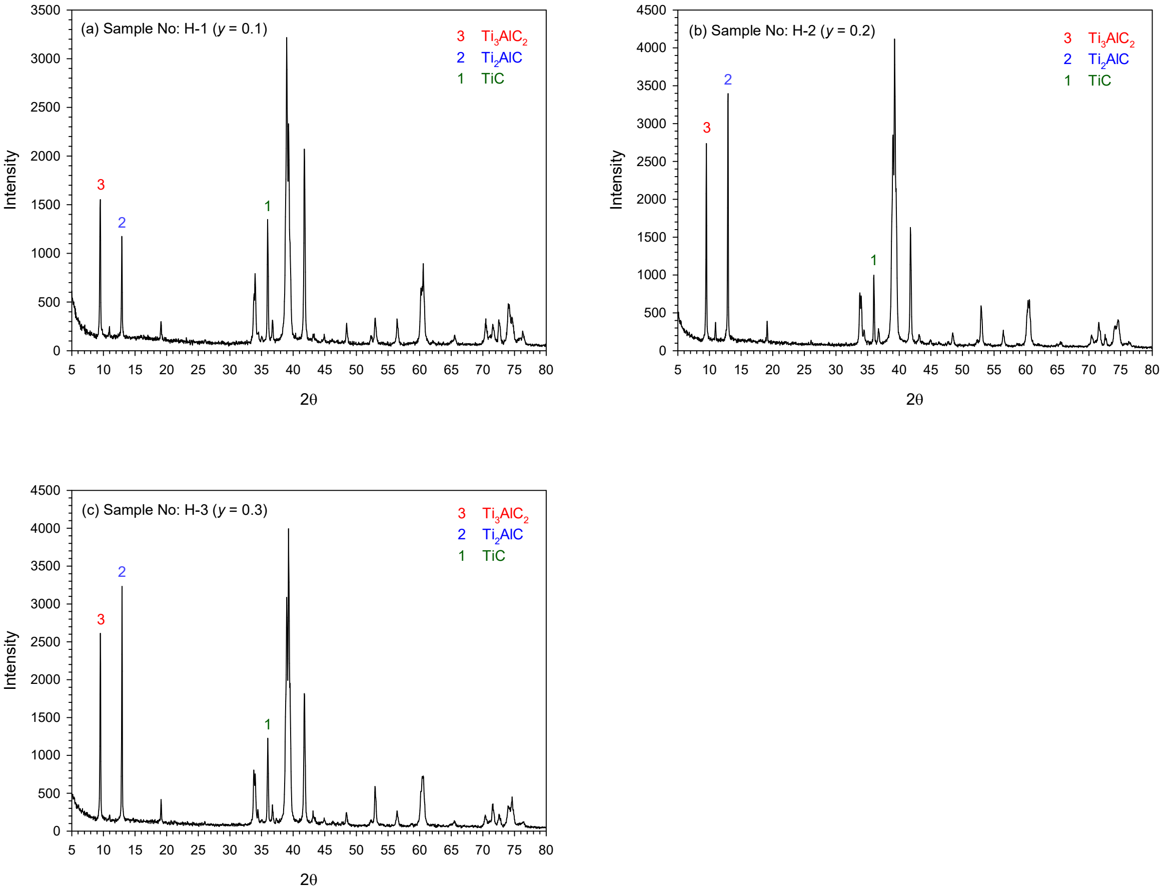

Figure S3. XRD patterns of synthesized products from (**a**) Sample H-1, (**b**) Sample H-2, and (**c**) Sample C-3.
